# Cardiometabolic syndrome among general adult population in Ghana: The role of lipid accumulation product, waist circumference‐triglyceride index, and triglyceride‐glucose index as surrogate indicators

**DOI:** 10.1002/hsr2.1419

**Published:** 2023-07-11

**Authors:** Enoch O. Anto, Joseph Frimpong, Wina I. O. Boadu, Emmanuel E. Korsah, Valentine C. K. T. Tamakloe, Ezekiel Ansah, Stephen Opoku, Emmanuel Acheampong, Evans A. Asamoah, Patience Nyarkoa, Eric Adua, Ebenezer Afrifa‐Yamoah, Max E. Annani‐Akollor, Christian Obirikorang

**Affiliations:** ^1^ Department of Medical Diagnostics, Faculty of Allied Health Sciences, College of Health Sciences Kwame Nkrumah University of Science and Technology Kumasi Ghana; ^2^ School of Medical and Health Sciences Edith Cowan University Perth Australia; ^3^ Department of Molecular Medicine, School of Medicine and Dentistry, College of Health Sciences Kwame Nkrumah University of Science and Technology Kumasi Ghana; ^4^ Department of Physiology, School of Medicine and Dentistry, College of Health Science Kwame Nkrumah University of Science and Technology Kumasi Ghana; ^5^ Rural Clinical School, Medicine and Health University of New South Wales Sydney New South Wales Australia; ^6^ School of Science Edith Cowan University Joondalup Australia

**Keywords:** anthropometric indices, cardiometabolic syndrome, insulin resistance, lipid accumulation product, triglyceride—glucose index, waist circumference—triglyceride index

## Abstract

**Background:**

Visceral obesity and insulin resistance contribute to developing cardiometabolic syndrome (MetS). We investigated the predictive abilities of lipid accumulation product (LAP), waist circumference‐triglyceride index (WTI), and triglyceride‐glucose (TyG) index for MetS screening among the general Ghanaian adults.

**Methods:**

The final prospective analysis included 4740 healthy adults aged 30–90 years from three communities comprising Ejisu, Konongo, and Ashanti Akim Agogo in Ghana. Self‐structured questionnaire pretested was used to collect sociodemographic, anthropometric, and clinical data. Blood samples were taken after fasting to measure glucose and lipid levels. LAP, WTI, and TyG were calculated from standard equations. MetS was defined by the International Diabetes Federation criteria. Receiver operating characteristic (ROC) curves and multivariable logistic regression were utilized to evaluate the potential of the three indices in identifying MetS.

**Results:**

Of the 4740 participants, 39.7% had MetS. MetS was more common in females (50.3%) than in males (22.2%). Overall, LAP ≥ 27.52 yielded as the best index for MetS with the highest area under the ROC curve (AUC) (0.866). At cut‐off LAP point of ≥23.87 in males and ≥33.32 in females, an AUC of 0.951 and 0.790 was identified in MetS prediction, respectively. LAP was an independent risk measure of MetS for both males (45.6‐fold) and females (3.7‐fold) whereas TyG was an independent risk measure for females (3.7‐fold) only.

**Conclusions:**

MetS is increasing among the general adult population. LAP and TyG are important sex‐specific risk measures to screen for MetS among the general adult population in our cohort.

## 1 INTRODUCTION

Metabolic syndrome (MetS) is a group of metabolic disorders, characterized by central obesity, hypertension, dysglycemia, high triglyceride (TG) levels, and low levels of high‐density lipoprotein cholesterol (HDL‐C).^1^, ^2^, ^3^ MetS is a major concern for public health worldwide and has been linked to higher risks of cardiovascular disease (CVD), type 2 diabetes, and mortality.^4^, ^5^ MetS occurrence ranges between 10% and 84% globally, and it is influenced by environmental factors, gender, ethnicity, and MetS classification criteria used.^6^, ^7^ In sub‐Saharan Africa, its prevalence is 18%^8^ and ranges from 12% to 31% in Ghana.^9^ The exact cause of MetS is not fully understood, but research suggests visceral obesity and insulin resistance (IR) as key factors of the condition.^10^ Visceral adiposity and IR are thus decent MetS predictors.

Magnetic resonance imaging and computed tomography are considered the standard methods for evaluating visceral adiposity,^11^ but are seldom utilized for routine investigations because of their high cost, radiation risks to patients, and laborious nature. Therefore, it is critical to develop a simple and clinically applicable visceral obesity surrogate indicator. As such, obesity indices are suggested to assess body fat levels and distribution, enabling monitoring of metabolic disorders. Body mass index (BMI), although being the most commonly used obesity index, has limitations in identifying fat distribution in the body.^12^ New indices have thus been proposed to assess visceral obesity. Among the recently proposed indices is lipid accumulation product (LAP). The LAP is a reliable indicator of central lipid buildup, and it was superior to BMI for predicting CVD risk.^13^ It is a combination of waist circumference (WC) and blood TGs.^13^ LAP is a valuable diagnostic tool for identifying IR, nonalcoholic fatty liver, and MetS, as evidenced by multiple studies.^13^, ^14^, ^15^

IR, another primary driving factor of MetS is diagnosed by hyperinsulinaemic‐euglycaemic clamp (HEC), a more complex and cumbersome method. Triglyceride‐glucose (TyG) and waist circumference‐triglyceride (WTI) indices are simple yet useful surrogate predictors of IR and have been shown as strong predictors for MetS in the Korean,^16^ United States,^17^ and the Chinese^18^, ^19^ populations.

Despite these novel findings and the usefulness of these measures in other populations, no study to the best of our knowledge, have simultaneously compared the value of these indices (LAP, WTI, and TyG) for screening MetS among healthy Ghanaian adults. It is in light of this that this study aimed to explore the potential of these three easily calculated and cheap indices as predictors of MetS among the general Ghanaian adult population.

## 2 MATERIALS AND METHODS

### 2.1 Study design/setting

This was a cross‐sectional study carried out across multiple centers from January 2022 to September 2022. We enrolled participants from three communities namely, Ejisu, Konongo, and Asante‐Akim Agogo in the Ashanti Region, Ghana. Initially, cities were split into geographic zones, and then these zones were further divided into smaller units called primary sampling units. These units were randomly chosen for further sampling. The participants were evaluated using a standardized procedures that included in‐person interviews and clinical exams. These evaluations were conducted at the study centers and followed standard protocols.

### 2.2 Study population and inclusion/exclusion criteria

A total of 5559 adults were initially recruited into the study. Healthy noninstitutionalized adults aged 30–90 years and residing in the Ejisu, Konongo, and Asante‐Akim Agogo communities were included in the study. Participants with known chronic illness and infections and were either attending medical check‐up or taking medications were excluded from the study. Other exclusion criteria were participants with missing data for anthropometric measurements (*n* = 199), blood pressure (*n* = 44), fasting plasma glucose (*n* = 23), or lipid levels (*n* = 121) and those aged below 30 years and above 90 years (*n* = 432) were excluded from this study. The final analysis included a total of 4740 adults.

### 2.3 Sample size estimation

With 7.4% MetS prevalence,^20^ expected 5% difference, and 0.05 type I error (*α*), 105 adults was the determined sample size. We extrapolated the population size to 4740 to increase the study's statistical power.

### 2.4 Ethical approval

Approval was sought from the Committee on Human Research, Publication and Ethics (CHRPE) at the School of Medical Sciences of the Kwame Nkrumah University of Science and Technology (KNUST), Ghana and we obtained written consent from every participant before the study began.

### 2.5 Data collection

Before recruiting participants, the purpose of the study was exaplained to them, and only those who consented to participate were included. A questionnaire was used to gather data on sociodemographic characteristics, including age and sex.

### 2.6 Blood pressure measurement

Trained nurses followed the American Heart Association (AHA) guidelines to measure blood pressure with a mercury sphygmomanometer and stethoscope.^21^ Two readings were taken, and the mean of the two, rounded to the nearest 2.0 mmHg, was documented.

### 2.7 Clinical and anthropometric measurements

#### 2.7.1 Blood sampling and biochemical analysis

Blood samples of 5 mL were collected from participants following an overnight fast. The serum separator tube contained 4 mL, and the fluoride‐oxalate tube contained 1 mL. After centrifugation, serum and plasma were stored at −80°C until assayed for FPG, HbA1C, TC, TG, and HDL‐C using the INTEGRA^(R)^ 400 plus Automated Chemistry Analyzer as per manufacturer's instructions (Fortress Diagnostics Ltd.). Low‐density lipoprotein cholesterol was estimated using the Friedewald formula.^22^

#### 2.7.2 Anthropometric measurements

Participants' weight (in kg) was measured using a bathroom scale (Zhongshan Camry Electronic Co. Ltd.) while wearing light clothing. To measure their height (in cm), a Seca 213 mobile stadiometer was used while they wore no shoes. Measuring tape was used to measure WC at the midpoint between the inferior angles of the ribs and the suprailiac crests. Hip circumference was measured at the widest point of the buttocks. Calculated indices included^19^, ^23^, ^24^, ^25^:

WHR  = waist (cm)/hip (cm),

WHtR  =  waist (cm)/height (cm),

$$\text{BMI}\;=\frac{\text{Weight}\;(\text{kg})}{\text{Height}\;{\left(m\right)}^{2}},$$

LAP =[WC (cm)–65]×TG (mmol/L) formalesand[WC (cm)–58]×TG (mmol/L)forfemale,

TyG  = Ln[TG (mg/dL)×FPG (mg/dL)/2],

WTI  = Ln[TG (mg/dL)×WC (cm)/2].

### 2.8 Definition of clinical terms

#### 2.8.1 Metabolic syndrome

Metabolic syndrome was defined by IDF criteria^26^ which involve the presence of central obesity (defined as BMI > 30 kg/m^2^) along with two of the following criteria: TG ≥ 150 mg/dL, low HDL‐C (<40 mg/dL for males and <50 mg/dL for females) or ongoing therapy for dyslipidaemia, BP ≥ 130/85 mmHg or ongoing therapy for hypertension, FPG ≥ 100 mg/dL or ongoing diabetes therapy.

#### 2.8.2 Data analysis

Data was entered into Microsoft Excel 2016, and SPSS v26 (SPSS Inc.) and GraphPad Prism 8.0.1 (GraphPad LLC) were utilized for analysis. Categorical data were expressed as frequency (proportion). Continuous data were checked for normality using Kolmogorov–Smirnov test. Nonparametric data were expressed as median (interquartile range). Nonparametric data was analyzed with Mann–Whitney *U*‐test to evaluate differences between groups, while *χ* ^2^ test was used to examine the relationship between sociodemographic characteristics (e.g., sex) and MetS. Receiver operating characteristic (ROC) curves and multivariable logistic regression were utilized to assess the potential of all three indices in identifying MetS. The cut‐off values for the indices generated on the ROC curves analysis were stratified into high and low and logistics regression analysis were performed. All statistical results obtained were deemed significant at *p* < 0.05.

## 3 RESULTS

### 3.1 Baseline characteristics of the study participants

At baseline, 1801/4740 (38.0%) males and 2938/4740 (62.0%) females were enrolled in the study. The female to male ratio for MetS prevalence was 4:1. The median age of participants was 55 years for the non‐MetS group and 57 years for the MetS group. Significantly higher median levels of FBS, HbA1c, TC, TG, VLDL‐C, coronary risk, SBP, and DBP were observed in subjects with MetS (all *p* < 0.0001). However, HDL‐C levels were significantly lower in MetS group than non‐MetS group. LDL‐C did not differ between the two groups. In addition, MetS group had significantly elevated anthropometric indices (LAP, TyG, and WTI) (all *p* < 0.0001) (Table 1).

**Table 1:** Baseline characteristics of subjects.

Variables	All (*N* = 4740)	No‐MetS (*N* = 2858)	MetS (*N* = 1882)	*p* Value
Sex				**<0.0001**
Male	1801 (38.0)	1400 (49.0)	401 (21.3)	
Female	2939 (62.0)	1458 (51.0)	1481 (78.7)	
Age (years)	56.00 (51.00–62.00)	55.00 (51.00–61.00)	57.00 (51.75–62.00)	0.280
FBS (mmol/L)	5.70 (5.40–6.30)	5.50 (5.30–5.90)	6.00 (5.70–6.60)	**<0.0001**
HbA1c (%)	5.20 (5.00–5.60)	5.10 (4.90–5.40)	5.40 (5.10–5.70)	**<0.0001**
TC (mmol/L)	4.50 (3.70–5.38)	4.40 (3.60–4.98)	4.60 (3.80–5.23)	0.895
TG (mmol/L)	1.11 (0.84–1.49)	0.98 (0.81–1.19)	1.47 (1.08–1.92)	**<0.0001**
HDL‐C (mmol/L)	1.20 (0.99–1.40)	1.30 (1.10–1.50)	1.10 (0.90–1.30)	**<0.0001**
LDL‐C (mmol/L)	2.68 (1.97–3.36)	2.73 (1.94–3.47)	2.60 (1.99–3.27)	0.504
Coronary risk	5.30 (4.43–6.25)	5.05 (4.21–5.88)	5.75 (4.85–6.88)	**<0.0001**
VLDL‐C (mmol/L)	0.50 (0.38–0.68)	0.44 (0.37–0.54)	0.67 (0.49–0.87)	**<0.0001**
Height (m)	1.62 (1.58–1.67)	1.63 (1.58–1.68)	1.61 (1.57–1.65)	0.106
Weight (kg)	67.30 (58.45–75.95)	62.50 (55.20–71.20)	74.35 (66.78–81.25)	**<0.0001**
SBP (mmHg)	143.00 (129.00–159.00)	138.00 (123.00–152.00)	151.00 (138.00–168.00)	**<0.0001**
DBP (mmHg)	85.00 (75.00–93.00)	82.00 (73.00–90.00)	89.00 (81.00–102.00)	**<0.0001**
WC (cm)	90.00 (81.50–98.00)	85.00 (76.00–92.00)	97.00 (91.75–102.00)	**<0.0001**
HC (cm)	101.00 (94.00–108.00)	96.00 (91.00–103.00)	106.00 (100.00–112.25)	**<0.0001**
WHR	0.89 (0.85–0.93)	0.87 (0.83–0.91)	0.92 (0.87–0.96)	**<0.0001**
BMI (kg/m^2^)	25.55 (22.18–29.03)	22.99 (21.00–26.49)	28.56 (25.71–31.60)	**<0.0001**
WHtR	0.56 (0.50–0.61)	0.51 (0.47–0.57)	0.60 (0.57–0.64)	**<0.0001**
LAP	32.55 (18.58–55.35)	21.00 (13.14–35.25)	56.07 (39.54–80.54)	**<0.0001**
TyG	8.54 (8.24–8.90)	8.36 (8.14–8.61)	8.87 (8.56–9.21)	**<0.0001**
WTI	8.36 (8.07–8.75)	8.18 (7.95–8.49)	8.75 (8.46–9.06)	**<0.0001**

*Note*: Categorical data are expressed as frequency (%), compared using *χ* ^2^ test. Nonparametric data are expressed as median (IQR); compared by Mann–Whitney test. All *p* less than 0.05 were deemed statistically significant. The bold value represents significant *p*‐values.

Abbreviations: BMI, body mass index; Coronary risk, TC/HDL‐C; DBP, diastolic blood pressure; FBS, fasting blood sugar; HbA1C, glycated hemoglobin; HC, hip circumference; HDL‐C, high‐density lipoprotein cholesterol; IQR, interquartile range; LAP, lipid accumulation product; LDL‐C, low‐density lipoprotein cholesterol; N, number; SBP, systolic blood pressure; TC, total cholesterol; TG, triglycerides; TyG, triglyceride glucose index; VLDL, very low‐density lipoprotein cholesterol; WC, waist circumference; WHR, waist‐to‐hip ratio; WHtR, waist‐to‐height ratio; WTI, waist triglyceride index.

### 3.2 Prevalence of MetS and its components in males and females

**Figure 1:**
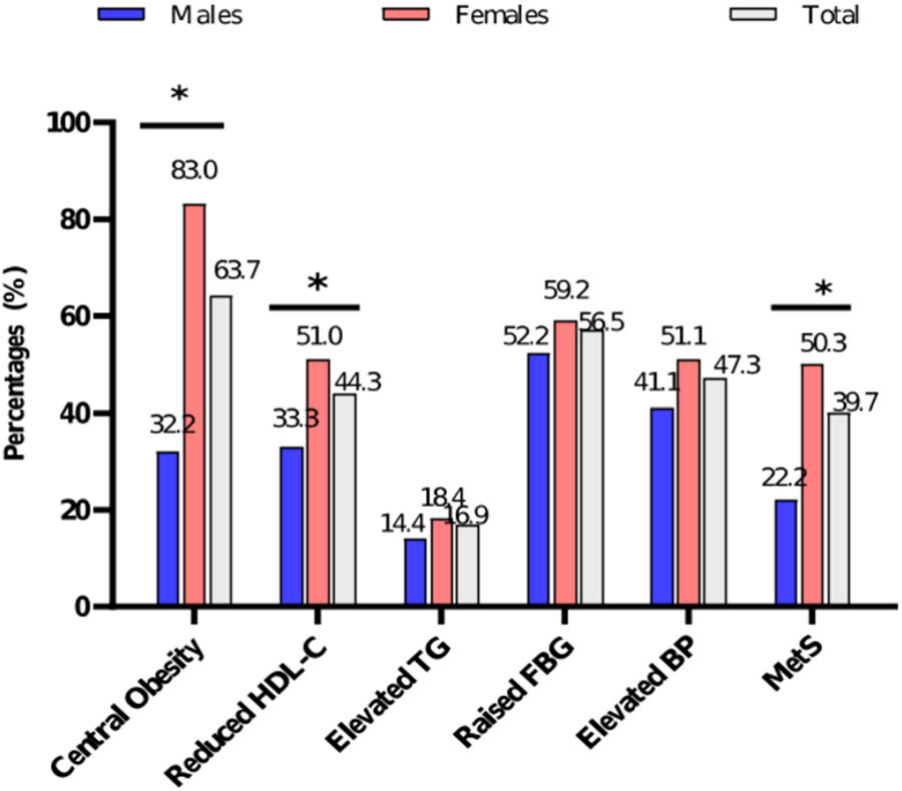
Prevalence of MetS and its components in males and females (*p < 0.001). BP, blood pressure; FBG, fasting blood glucose; HDL‐C, high‐density lipoprotein cholesterol; MetS, cardiometabolic syndrome; TG, triglyceride.

1881/4740 (39.7%) of the participants were diagnosed with MetS. MetS and its components were more prevalent in females than males. Additionally, MetS varied significantly between male and female subjects. Of the individual MetS components, only central obesity and reduced HDL‐C differed between both sexes (both *p* < 0.0001) (Figure 1).

### 3.3 The anthropometric indices for predicting MetS

Table 2 shows the diagnostic performance of LAP, TyG, and WTI for MetS. For males, the optimum cut‐off points of LAP identified were ≥23.87 (sensitivity: 0.95, specificity: 0.84) and for females, the optimum cut‐off points of LAP were ≥33.32 (sensitivity: 0.84, specificity: 0.60). For males, the ideal WTI cut‐off was ≥8.58, while for females, it was ≥8.56. Table 2 displays additional information including negative predictive value and positive predictive value.

**Table 2:** The anthropometric indices for predicting MetS.

	AUC	95% CI (lower)	95% CI (upper)	Cut‐off	Sensitivity	Specificity	PPV	NPV
Males								
LAP	0.951	0.912	0.991	≥23.87	0.95	0.84	0.63	0.98
WTI	0.893	0.807	0.978	≥8.58	0.80	0.90	0.70	0.94
TyG	0.857	0.759	0.955	≥8.59	0.89	0.79	0.55	0.96
Females								
LAP	0.790	0.718	0.862	≥33.32	0.84	0.60	0.68	0.78
WTI	0.765	0.688	0.842	≥8.56	0.68	0.79	0.77	0.70
TyG	0.761	0.682	0.840	≥8.74	0.68	0.80	0.78	0.70
Overall								
LAP	0.866	0.821	0.911	≥27.52	0.90	0.68	0.65	0.92
WTI	0.816	0.759	0.873	≥8.54	0.72	0.82	0.72	0.82
TyG	0.794	0.733	0.855	≥8.70	0.71	0.81	0.72	0.80

Abbreviations: AUC, area under curve; CI, confidence interval; LAP, lipid accumulation products; NPV, negative predictive value; PPV, positive predictive value; TyG, triglyceride glucose index; WTI, waist circumference triglyceride index.

### 3.4 ROC of LAP, TyG, and WTI for identifying MetS

**Figure 2:**
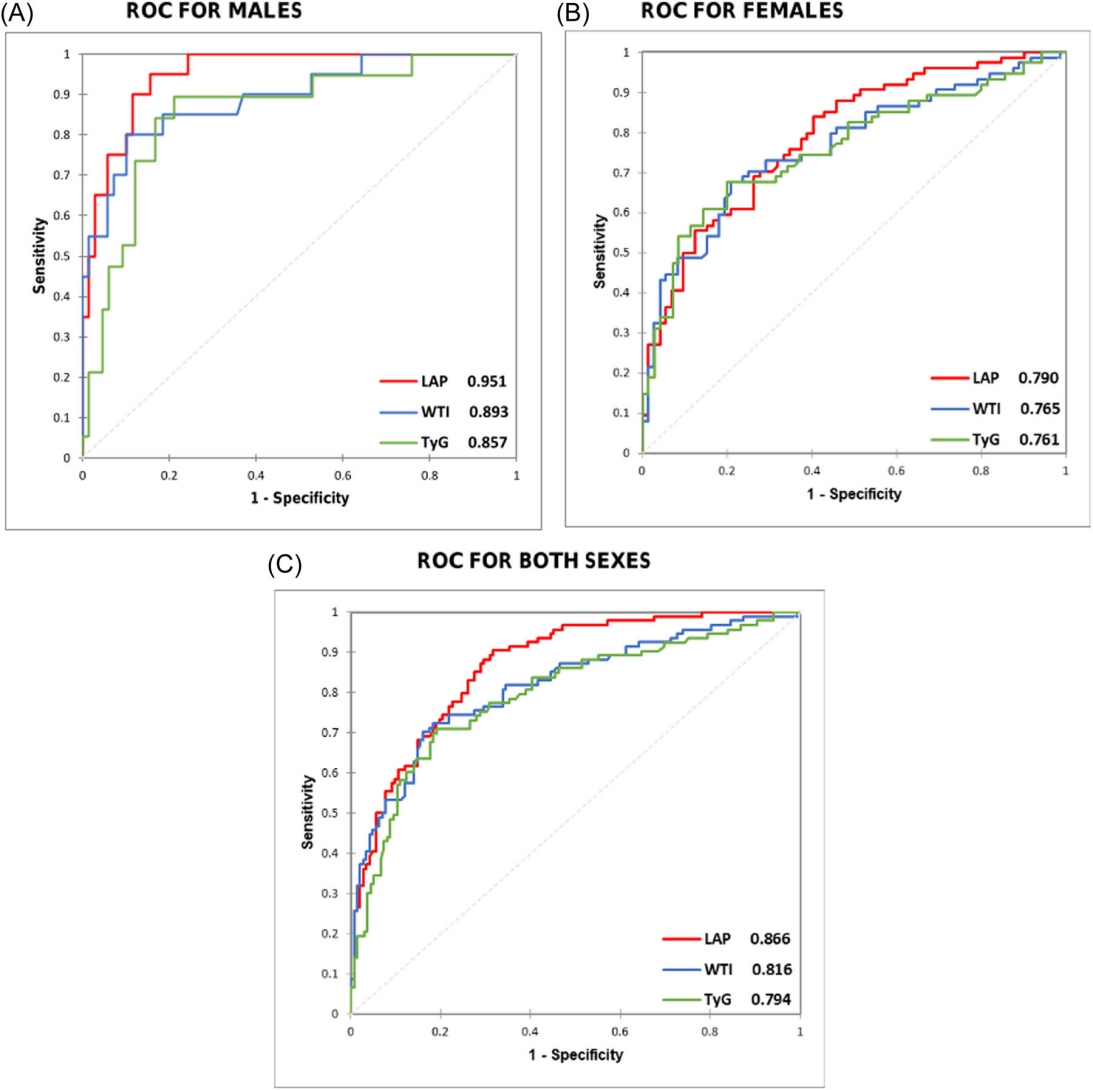
Receiver operating characteristic curve (ROC) of LAP, WTI, and TyG for identifying MetS. (A) ROC for females; (B) ROC for males; and (C) ROC for both sexes. LAP, lipid accumulation product; TyG, triglyceride‐glucose; WTI, waist circumference‐triglyceride index.

Figure 2 shows the ROC of LAP, TyG, and WTI indicating the discriminating power (AUC) for MetS identification. Overall, LAP yielded as the best index for predicting MetS followed by WTI and then TyG. At a cut‐off value of ≥23.87 for males and ≥33.32 for females, LAP yielded an AUC of 0.951 (95% confidence interval [95% CI]: 0.912–0.991) and 0.790 (95% CI: 0.718–0.862) for predicting MetS, respectively (Figure 2). At a cut‐off value of ≥8.58 for males and ≥8.56 for females, WTI yielded an AUC of 0.893 (95% CI: 0.807–0.978) and 0.765 (95% CI: 0.688–0.842) for predicting MetS, respectively.

### 3.5 Comparison of AUC values of the indices

For males, the AUC values for LAP and TyG were significantly different (*p* = 0.0153) whereas that between LAP and WTI was not significant. This shows that LAP was a stronger predictor for MetS than the other indices.

AUC values for females did not significantly differ between LAP, TyG, and WTI (all *p* values > 0.05) (Table 3).

**Table 3:** Comparison of AUC values for the three indices.

	Difference between area	*p* Value
Males		
LAP versus WTI	0.0582	0.0557
LAP versus TyG	0.0942	**0.0153**
Females		
LAP versus WTI	0.0251	0.1513
LAP versus TyG	0.0294	0.3146

*Note*: Compared using Delong's nonparametric method. The bold value represents significant *p* values.

Abbreviations: LAP, lipid accumulation product; TyG, triglyceride‐glucose; WTI, waist circumference‐triglyceride index.

### 3.6 Logistic regression of anthropometric indices for predicting MetS in males and females

After adjusting for age, males with LAP ≥ 23.87 were 45.6 times increased odds of having MetS compared to those with low LAP levels (<23.87).

In the adjusted model for females, the higher risk group of LAP (LAP ≥ 33.12) and TyG (TyG ≥ 8.74) were at 3.7 times increased odds of having MetS compared to those in the lower risk group respectively, adjusted odds ratio (aOR) = 3.67 (1.42–9.54), *p* = 0.007 versus aOR = 3.66 (1.10–12.23), *p* = 0.035.

LAP was the independent predictor for MetS in both males and females whereas TyG was the independent predictor of MetS in females only (Table 4).

**Table 4:** Associations between MetS incidence and the indices.

Indices	cOR (95%CI)	*p* Value	aOR (95%CI)^a^	*p* Value
*Males*				
LAP				
<23.87	Ford et al.^1^		Ford et al.^1^	
≥23.87	101.91 (12.34–841.74)	**<0.0001**	45.61 (3.97–524.51)	**0.002**
TyG				
<8.59	Ford et al.^1^		Ford et al.^1^	
≥8.59	19.81 (5.05–77.73)	**<0.0001**	1.16 (0.10–13.08)	0.904
WTI				
<8.58	Ford et al.^1^		Ford et al.^1^	
≥8.58	27.00 (7.52–96.95)	**<0.0001**	3.09 (0.37–25.98)	0.298
*Females*				
LAP				
<33.32	Ford et al.^1^		Ford et al.^1^	
≥33.32	7.66 (3.52–16.66)	**<0.0001**	3.67 (1.42–9.54)	**0.007**
TyG				
<8.74	Ford et al.^1^		Ford et al.^1^	
≥8.74	7.84 (3.67–16.74)	**<0.0001**	3.66 (1.10–12.23)	**0.035**
WTI				
<8.56	Ford et al.^1^		Ford et al.^1^	
≥8.56	7.45 (3.54–15.69)	**<0.0001**	1.26 (0.34–4.66)	0.725

*Note*: Comparison by univariable and multivariable logistic regression; *p* values less than 0.05 were deemed significant. Bold value represents significant *p* values.

Abbreviations: aOR, adjusted odds ratio; cOR, crude odds ratio; LAP, lipid accumulation products; TyG, triglyceride‐glucose index; WTI, waist circumference‐triglyceride index.

^a^ Adjusted for age.

## 4 DISCUSSIONS

In this study, explored the potential of three easily calculated and cost‐effective obesity indices—LAP, WTI, and TyG index—as predictors of MetS among the Ghanaian healthy adult population. Among the healthy adult population, 39.7% were screened with MetS, being more prevalent in females (50.3%) compared to males (22.2%). Our study also revealed that LAP, WTI, and TyG could predict subjects with MetS per the ROC curve analysis: Their AUC values exceeded 0.70 in both sexes. LAP showed the highest diagnostic potential, followed by WTI, and then TyG. However, only LAP could independently predict MetS in both sexes after age was adjusted for in the multivariable logistic regression. TyG could independently predict MetS in females only. This suggests that LAP and TyG may be useful sex‐specific indices for MetS prediction.

The current study found a significant proportion (39.7%) of the participants to have MetS. However, comparing the prevalence of MetS in different studies is difficult because of the varied definitions used in the literature. Compared to this current study, several studies conducted in different regions, such as the United States,^27^ Central America,^28^ and Argentina^29^ have reported lower prevalence rates of MetS, ranging from 31% to 37%. However, Crowther and colleagues found a slightly higher prevalence (42%) among South Africans^30^ compared to the current study (39.7%). It is not unexpected to find a high prevalence of MetS in this current study, given that the factors that constitute MetS, namely elevated blood pressure, central obesity, and fasting blood glucose, are highly prevalent among the subjects (Figure 1). The high occurrence of MetS has been associated with urbanization, western lifestyle, changes in diet, and disease patterns. Therefore, it is important for policymakers and healthcare managers to take prompt action and prioritize regular screening for all MetS components at the primary healthcare level. In addition, MetS was more prevalent in females (50.3%) than males (22.2%) in the current study. This finding is similar to several previous studies across different populations.^31^, ^32^, ^33^ This difference in prevalence is not surprising because MetS is a sex‐specific condition resulting from multiple interconnected risk factors.^33^ But we also believe that the much higher prevalence in females than males could be due to the higher females than males in the current study (female: male ratio was 4:1).

Our study also reveals LAP as the best measure to discriminate MetS among the subjects as supported by our ROC analysis and logistic regression analysis: LAP showed the highest AUC of 0.951, 0.790, and 0.866 for males, females, and both sexes respectively; it could also independently predict MetS among the subjects in the adjusted logistic regression model. In this study, LAP proved “outstanding” (AUC ≥ 0.9) for MetS prediction in males but proved “excellent” (0.8 ≤ AUC < 0.9) in females per Homer and Lemeshow's criteria^34^ for AUC. In keeping with our results, Sun et al.^17^ found LAP as the index with the highest diagnostic potential for MetS in United States adults (overall AUC: 0.857). In their study, however, they included visceral adiposity index (VAI) in their comparison. Likewise, Shin et al.^16^ found LAP as the best index for MetS prediction among Korean adults (overall AUC: 0.917). In their study too, they included both VAI and waist‐to‐height ratio (WHtR) in their comparison. A possible explanation for LAP's strong predictive ability for MetS across these different studies could be that LAP is linked to lipolytic and dysfunctional adipose tissue, which is a major contributor to MetS and its accompanying conditions such as CVDs and T2DM.^35^ Likewise, LAP components—WC and TG—are potent predictors of MetS, CVDs, and T2DM on their own. WC, one component of LAP, measures abdominal fat, including subcutaneous and visceral fat. High WC strongly predicts cardiometabolic diseases and is a core component of MetS.^36^ TG, the other component of LAP, which is a significant contributor to MetS, is more closely associated with IR, compared to HOMA‐IR.^37^

Furthermore, the optimal cut‐off of LAP for predicting MetS in this study was ≥23.87 for males and ≥33.32 for females. Much higher cut‐off values of LAP were observed among the US adults (≥53.31 for males and ≥52.43 for females),^17^ the Brazilians (≥64.1 for males and ≥38.0 for females),^15^ and the Argentinians (≥53.63 for males and ≥53.63 for females).^38^ The variations in the optimal cut‐offs observed for the different populations could be due to the differences in ages of the recruited group, variation in IR and abdominal fat distribution, and the MetS diagnostic criteria adopted.

Our next finding that TyG could be a better predictor of MetS in females only is supported by our ROC and logistic regression analysis: TyG yielded an AUC of 0.857, 0.761, and 0.794 for males, females, and both sexes, respectively; it could independently predict MetS among only female subjects in the adjusted logistic regression model. We found that TyG was “excellent” (0.8 ≤ AUC < 0.9) for MetS prediction in males but it was “acceptable” (0.7 ≤ AUC < 0.8) in females per Hosmer and Lemeshow's criteria for AUC. This signifies that TyG could be useful for MetS prediction in female subjects, though, weaker than LAP.

TyG index has been linked to an increased risk of cardiovascular events.^39^, ^40^, ^41^ In contrast to our findings, several studies have confirmed TyG's ability to independently identify MetS in both sexes. In Chinese adults, the authors suggested TyG as an independent predictor of MetS with 0.802 overall AUC, and optimal cut‐off points of 8.9 and 8.7 in men and women, accordingly.^42^ In Korean adults, Shin et al. found similar results.^42^ In US adults, the authors reported 0.828 overall AUC, and best cut‐off points of 8.8 and 8.7 in men and women accordingly.^17^ In our current study, the best cut‐off points in men (8.6) and women (8.7), were consistent with the earlier findings. We believe that a possible reason why TyG failed to independently discriminate MetS among male subjects in the current study could be due to the significant difference in AUC between LAP and TyG in males (Table 3). This suggests that LAP was stronger than TyG in identifying MetS and it could have influenced its ability after we adjusted for age in the multivariate logistic regression. However, AUC values for females did not significantly differ between LAP and TyG in identifying MetS. Sun and colleagues^17^ reported a similar finding.

Our study revealed WTI as the least potent of all the three indices studied: it yielded an AUC of 0.893, 0.765, and 0.816 for males, females, and both sexes respectively but failed to independently predict MetS in both sexes after age was controlled.

In contrast to our finding, Liu et al., who first proposed WTI found it to be closely related to MetS, with an “excellent” (0.8 ≤ AUC < 0.9) AUC for MetS prediction in both male and female Chinese population.^19^ Sun et al. reported similar findings in the US population.^17^ The usefulness of WTI for predicting MetS could be that like LAP, WTI combines WC and TG in its computation (WC and TG are key components of MetS). Furthermore, AUC values for LAP and WTI did not differ significantly in both sexes in our study and we found optimal cut‐off values of WTI to be 8.58 and 8.56 for males and females, respectively. However, in Sun et al.'s study, the difference in AUC values between LAP and WTI was significant in both sexes and they found optimal cut‐off values of WTI to be 8.88 and 8.56 for males and females, respectively.^17^ The disparity in these findings could be due to ethnic differences since these indices are ethnic‐specific. MetS criteria adopted could have also accounted for this disparity. We adopted the International Diabetes Federation (IDF) 2005 criteria whereas the previous study adopted that of NCEP‐ATP III for Americans.

An advantage of this study is that it is the first of its kind to be conducted in the Ghanaian population. This study had limitations noteworthy to mention. First, our study is a cross‐sectional study and as such conclusions may change over time. A future prospective study is required to evaluate how the present findings affect MetS. Second, only Ghanaians were recruited and so results may not apply to other nationalities. Finally, this study adopted IDF 2005 criteria for MetS diagnosis. Thus, whether other MetS criteria will yield reproducible results requires further investigation.

In conclusion, we explored the potential of three easily calculated and cheap obesity and lipid‐related indices namely LAP, TyG, and WTI in identifying MetS in apparently healthy Ghanaian adults. LAP yielded as the best marker for MetS with the highest AUC and was an independent predictor of MetS in both males and females. However, TyG was an independent predictor of MetS in females only. MetS is becoming more common among the healthy adult population. LAP and TyG are important gender‐specific risk measures to screen for MetS among the apparently healthy population in our cohort.

## AUTHOR CONTRIBUTIONS

**Enoch O. Anto**: Conceptualization; Data curation; Investigation; Methodology; Project administration; Supervision; Writing—review and editing. **Joseph Frimpong**: Conceptualization; Data curation; Formal analysis; Investigation; Methodology; Writing—review and editing. **Wina I. O. Boadu**: Conceptualization; Investigation; Methodology; Supervision; Writing—review and editing. **Emmanuel E. Korsah**: Formal analysis; Investigation; Writing—original draft; Writing—review and editing. **Valentine C. K. T. Tamakloe**: Investigation; Software; Visualization; Writing—review and editing. **Ezekiel Ansah**: Data curation; Formal analysis; Software; Writing—original draft; Writing—review and editing. **Stephen Opoku**: Formal analysis; Methodology; Writing—original draft; Writing—review and editing. **Emmanuel Acheampong**: Investigation; Methodology; Resources; Writing—review and editing. **Evans A. Asamoah**: Formal analysis; Methodology; Writing—original draft; Writing—review and editing. **Patience Nyarkoa**: Methodology; Writing—review and editing. **Eric Adua**: Methodology; Writing—review and editing. **Ebenezer Afrifa‐Yamoah**: Investigation; Methodology; Supervision; Writing—review and editing. **Max E. Annani‐Akollor**: Methodology; Supervision; Writing—review and editing. **Christian Obirikorang**: Methodology; Supervision; Writing—review and editing.

## CONFLICT OF INTEREST STATEMENT

The authors declare no conflict of interest.

## TRANSPARENCY STATEMENT

The lead author Enoch Odame Anto affirms that this manuscript is an honest, accurate, and transparent account of the study being reported; that no important aspects of the study have been omitted; and that any discrepancies from the study as planned (and, if relevant, registered) have been explained.

## Data Availability

Data are available upon request from corresponding author.
